# Late-Detected Sinus Venosus Atrial Septal Defect and Subsequent Enteroviral Central Nervous System Infection: A Case Report

**DOI:** 10.7759/cureus.116219

**Published:** 2026-09-14

**Authors:** Aoumar G Chamma, Wendy Saliba, Linda Chamma

**Affiliations:** 1 Cardiology, University of Balamand, Beirut, LBN; 2 Urology, Lebanese University, Beirut, LBN

**Keywords:** adult congenital heart disease, enteroviral central nervous system infection, partial anomalous pulmonary venous return, persistent left superior vena cava, postoperative right ventricular dysfunction, sinus venosus atrial septal defect, transient loss of consciousness

## Abstract

Sinus venosus atrial septal defect (SVASD) is frequently associated with partial anomalous pulmonary venous return and may be missed on routine transthoracic echocardiography because of its superior and posterior location. We report a 68-year-old woman with atrial fibrillation who presented with dyspnea and marked right-sided chamber enlargement. Cardiac magnetic resonance imaging identified a 33 × 21-mm superior SVASD, anomalous drainage of the right upper and middle pulmonary veins, and a persistent left superior vena cava. The Qp/Qs was 3.7 by cardiac magnetic resonance and 3.2 by right heart catheterization, with a low indexed pulmonary vascular resistance of 1.7 Wood units·m², supporting surgical repair. Three and a half months after successful repair, the patient presented with transient loss of consciousness. Cardiac evaluation showed no residual interatrial shunt, no intracardiac thrombus, and no significant arrhythmia, although right ventricular dysfunction persisted. Brain magnetic resonance imaging demonstrated asymmetric cortical and deep gray matter abnormalities. Cerebrospinal fluid showed lymphocytic pleocytosis, mildly elevated protein, preserved glucose, and positive enterovirus polymerase chain reaction. These findings supported enteroviral central nervous system infection. Because atrial fibrillation and postoperative cardiac abnormalities provided competing explanations for the neurologic presentation, the exact mechanism of the transient loss of consciousness remained uncertain. This case illustrates the importance of avoiding diagnostic anchoring when new neurologic symptoms develop after congenital cardiac surgery.

## Introduction

Sinus venosus atrial septal defect (SVASD) is an uncommon atrial-level communication frequently associated with partial anomalous pulmonary venous return (PAPVR) [1-4]. Because of its high posterior location, it may be missed on routine transthoracic echocardiography, and multimodality imaging may be required to define the defect and associated venous anatomy [2,3,5]. In older patients, assessment of shunt magnitude and pulmonary vascular resistance is important when considering repair [1,2,6].

We report a 68-year-old woman with a previously unrecognized large SVASD and PAPVR who underwent surgical repair. Three and a half months later, she presented with transient loss of consciousness, prompting evaluation for postoperative cardiac, thromboembolic, and neurologic causes. Enterovirus was subsequently detected in cerebrospinal fluid. The temporal sequence does not establish causality between the cardiac operation and the central nervous system infection but illustrates the diagnostic challenge of evaluating new neurologic symptoms after congenital cardiac surgery.

## Case presentation

A 68-year-old woman with hypertension and atrial fibrillation treated with apixaban had previously been asymptomatic and reported excellent functional capacity. She had no meaningful limitation in her daily activities and had completed four pregnancies without recognized cardiac complications. Her long asymptomatic course was notable given the size of the congenital shunt that was later identified and likely reflected gradual right ventricular adaptation over several decades.

Initial transthoracic echocardiography demonstrated marked right atrial and right ventricular enlargement, but no atrial septal defect was visualized. The unexplained right-sided volume overload prompted further evaluation for an occult intracardiac shunt (Figure 1).

**Figure 1:**
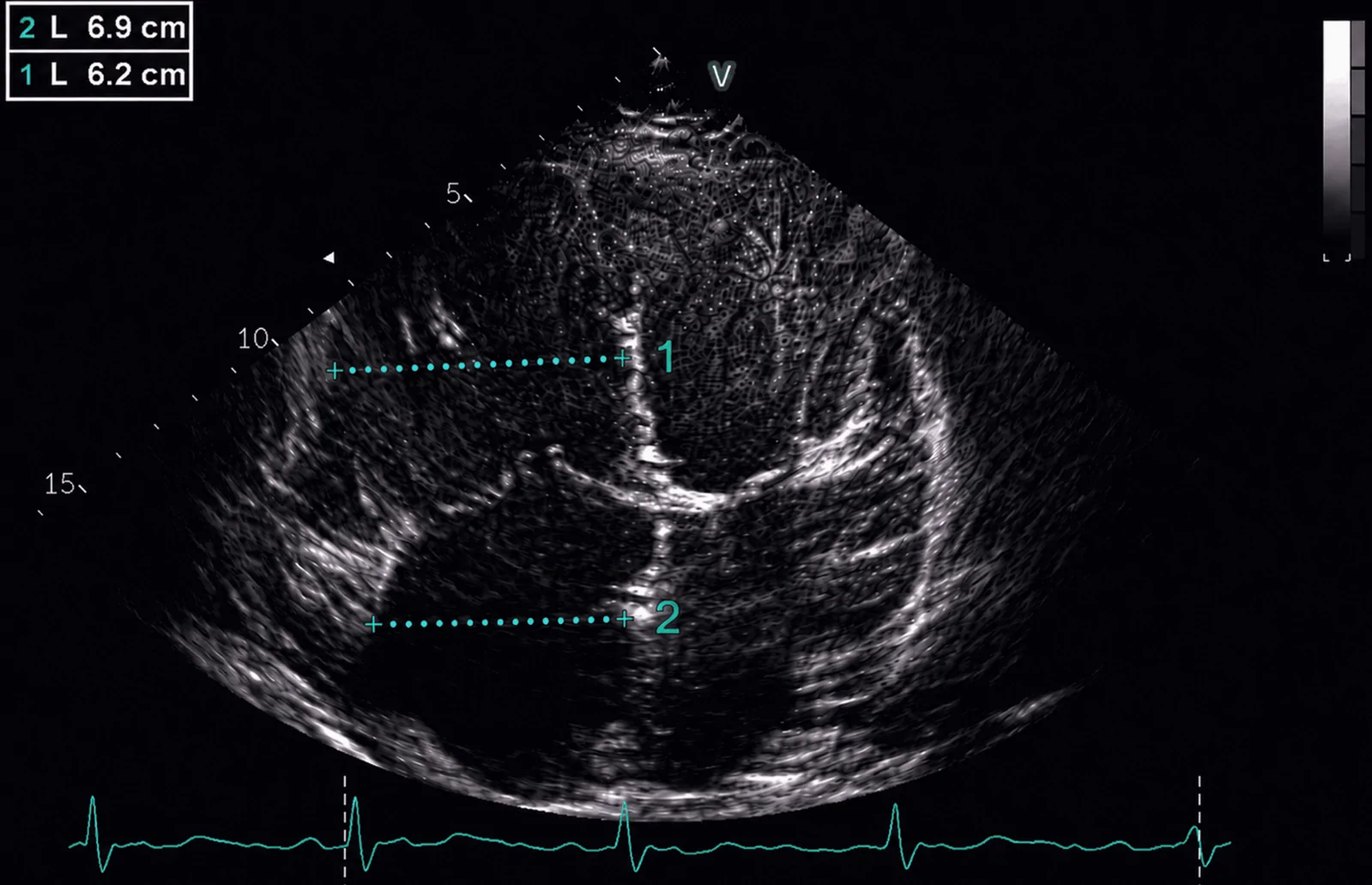
Initial apical four-chamber transthoracic echocardiographic view showing marked right ventricular and right atrial dilatation, with right ventricular basal diameter measuring 6.9 cm and right atrial transverse diameter measuring 6.2 cm.

Cardiac magnetic resonance imaging (CMR) was subsequently performed and demonstrated a large superior SVASD measuring 33 × 21 mm. The communication was located high and posteriorly near the superior vena cava-right atrial junction, which explained why it had not been identified on the initial routine transthoracic examination (Figure 2).

**Figure 2:**
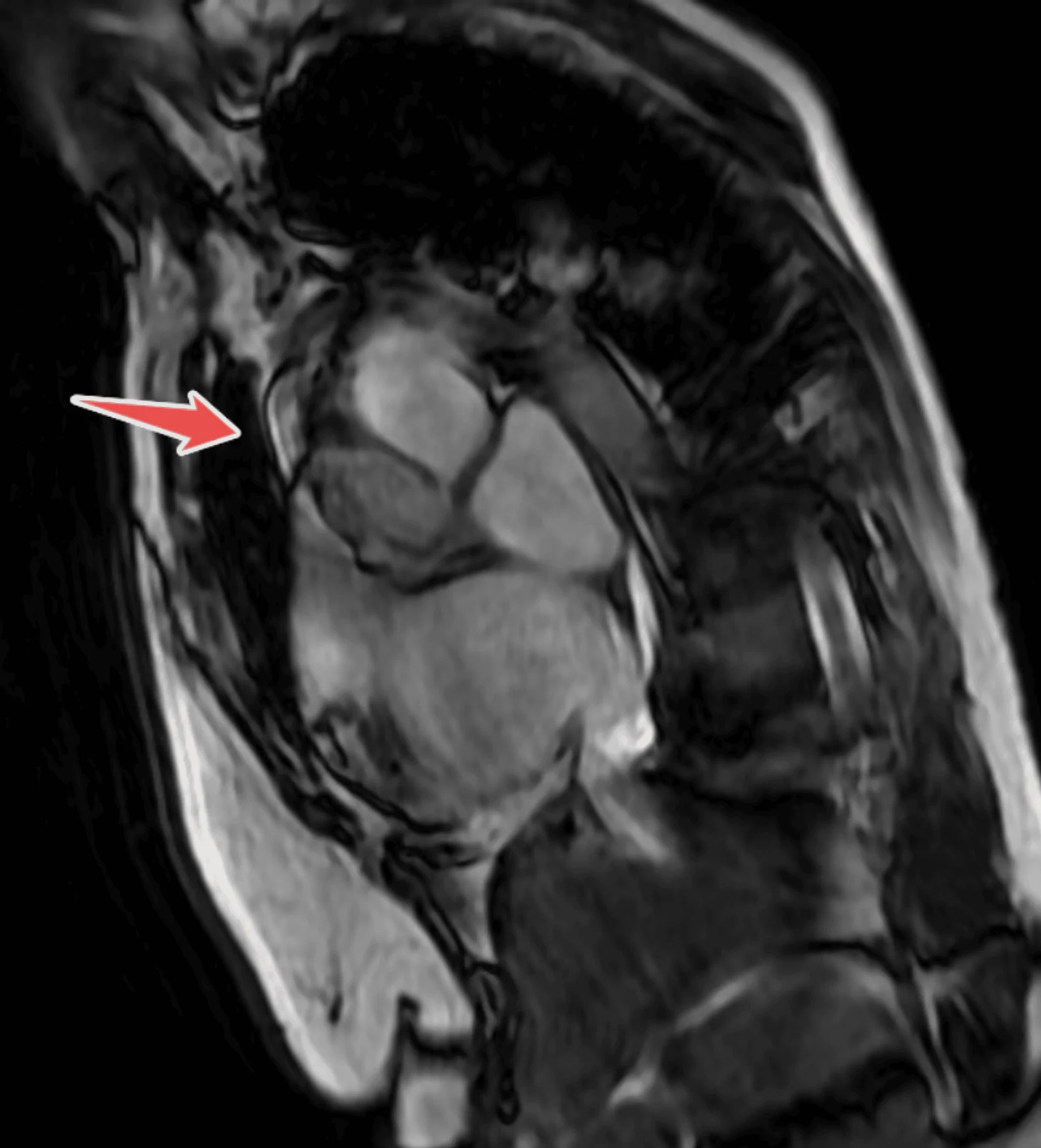
Preoperative cine cardiac magnetic resonance imaging showing a superior sinus venosus atrial septal defect at the superior vena cava-right atrial junction, highlighted by an arrow.

CMR also demonstrated PAPVR, with the right upper pulmonary vein draining into the superior vena cava and the right middle pulmonary vein near the superior vena cava-right atrial junction. The Qp/Qs was 3.7, confirming a large left-to-right shunt. The right ventricle was severely dilated but retained preserved systolic function with an ejection fraction of 62%. A persistent left superior vena cava draining through the coronary sinus was also identified (Figure 3).

**Figure 3:**
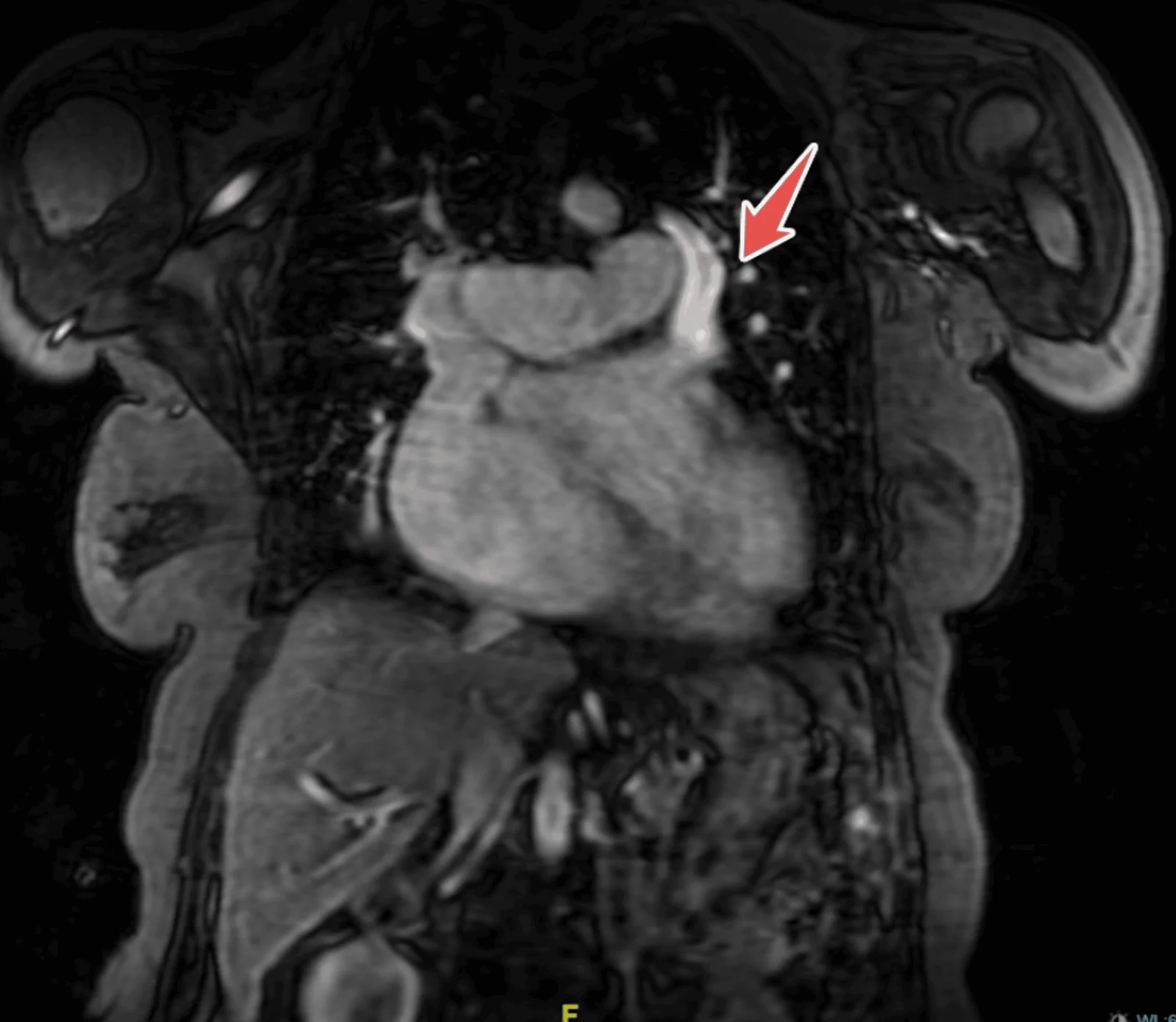
Coronal contrast-enhanced magnetic resonance angiography showing a persistent left superior vena cava descending along the left mediastinum, highlighted by an arrow.

Right heart catheterization was performed to assess the magnitude of the shunt, pulmonary hemodynamics, and operability. The findings are summarized in Table 1.

**Table 1:** Preoperative right heart catheterization findings. Qp/Qs: pulmonary-to-systemic blood flow ratio

Parameter	Result
Right atrial pressure	14 mmHg
Right ventricular pressure	43/22 mmHg
Pulmonary artery pressure	40/24 mmHg
Mean pulmonary artery pressure	33 mmHg
Pulmonary capillary wedge pressure	17 mmHg
Superior vena cava oxygen saturation	85%
Inferior vena cava oxygen saturation	75%
Right atrial oxygen saturation	92%
Right ventricular oxygen saturation	88%
Pulmonary artery oxygen saturation	87.80%
Systemic arterial oxygen saturation	95%
Qp/Qs	3.2
Indexed pulmonary vascular resistance	1.7 Wood units·m²

Right heart catheterization confirmed a large left-to-right shunt, with a Qp/Qs of 3.2, a mean pulmonary artery pressure of 33 mmHg, and a low indexed pulmonary vascular resistance of 1.7 Wood units·m², supporting operability. The modest difference from the CMR-derived Qp/Qs of 3.7 did not alter the clinical interpretation. A focused preoperative transthoracic echocardiogram confirmed the atrial-level left-to-right shunt and showed a severely dilated right ventricle with preserved systolic function and mild tricuspid regurgitation. Continuous-wave Doppler across the tricuspid valve demonstrated a peak velocity of 2.74 m/s, corresponding to a maximum tricuspid regurgitation pressure gradient of approximately 30 mmHg and an estimated systolic pulmonary artery pressure of approximately 35 mmHg (Figure 4).

**Figure 4:**
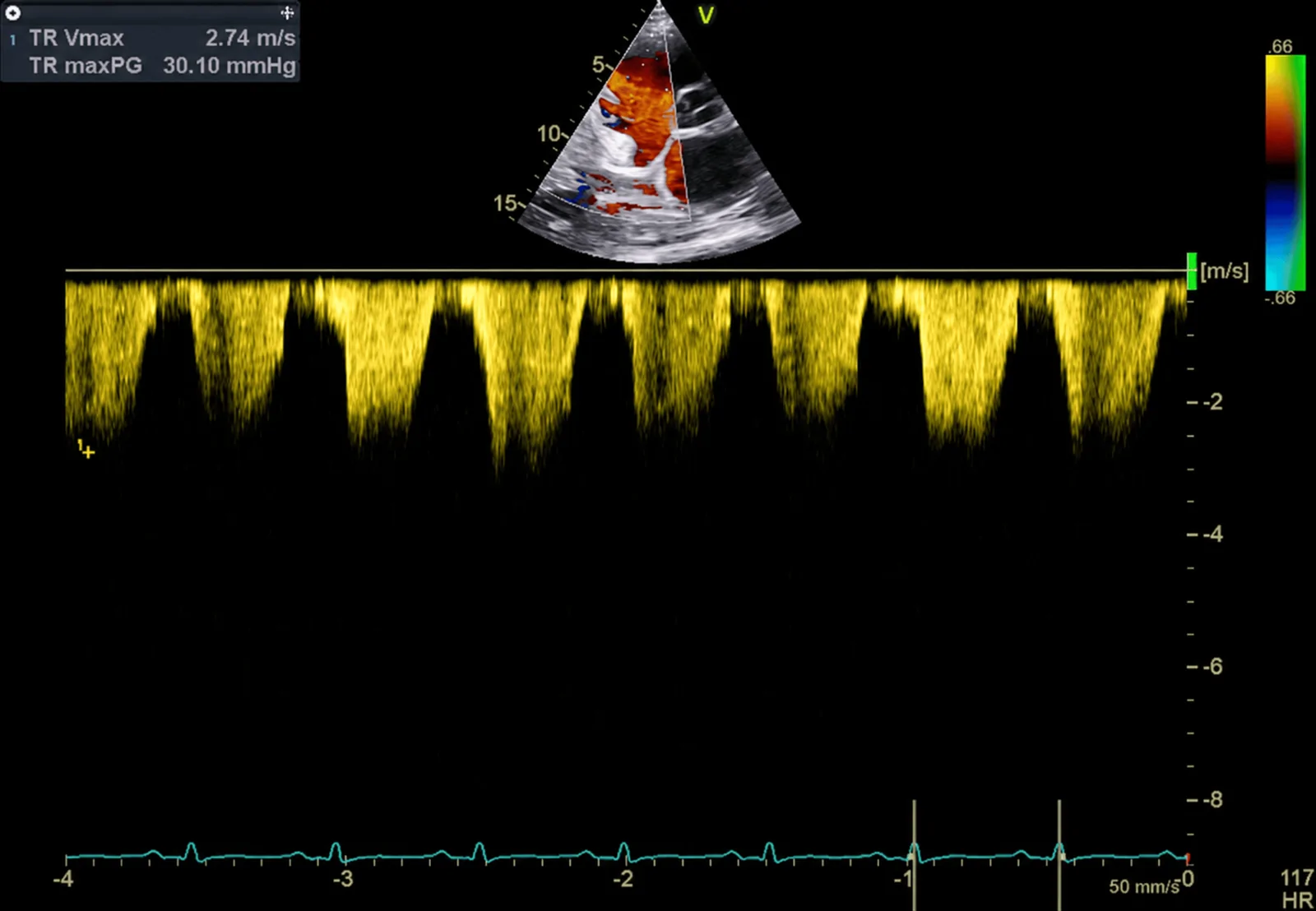
Parasternal short-axis view with preoperative tricuspid-valve continuous-wave Doppler tracing showing a peak velocity of 2.74 m/s.

Preoperative coronary angiography showed no significant obstructive coronary artery disease. The patient subsequently underwent surgical repair with patch closure of the SVASD and rerouting of the anomalous right pulmonary veins toward the left atrium. The persistent left superior vena cava draining into the coronary sinus was left intact. Anticoagulation for atrial fibrillation was resumed postoperatively.

Early postoperative TTE showed no residual interatrial shunt and preserved left ventricular systolic function. The right ventricle remained severely dilated with reduced systolic function, moderate tricuspid regurgitation, and an estimated systolic pulmonary artery pressure of approximately 35 mmHg.

Three and a half months after surgical repair, the patient presented to the emergency department after a single episode of transient loss of consciousness. The event occurred while she was sitting and was preceded by nausea. According to a witness, she became unresponsive for approximately 40-50 seconds, without tonic-clonic movements, other abnormal motor activity, tongue biting, urinary incontinence, or traumatic injury. She regained consciousness spontaneously and experienced brief confusion lasting approximately two minutes before returning to her baseline neurologic status. No persistent focal neurologic deficit was observed, and she had experienced no similar or recurrent episodes before admission.

During the preceding seven days, she had no fever, headache, vomiting, diarrhea, upper respiratory symptoms, or generalized malaise. She also denied neck stiffness, photophobia, rash, focal weakness, or speech disturbance. No recent travel, known sick contact, exposure to children with an acute illness, or other specific infectious exposure was identified. The absence of a classical meningeal syndrome made a central nervous system infection less apparent at presentation, particularly because transient loss of consciousness was the dominant clinical feature. Given her recent cardiac surgery, atrial fibrillation, persistent right-sided cardiac remodeling, right ventricular dysfunction, and valvular regurgitation, the event initially prompted evaluation for both cardiac and noncardiac causes of transient loss of consciousness.

Electrocardiography showed atrial fibrillation with right-axis deviation and no acute ST-segment changes. Troponin was negative, arguing against acute myocardial injury. N-terminal pro-B-type natriuretic peptide (NT-proBNP) was elevated at 3897 pg/mL, consistent with ongoing myocardial and right-sided hemodynamic stress. During hospitalization, no sustained ventricular arrhythmia, high-grade atrioventricular block, or clinically significant pauses were documented on cardiac monitoring.

Repeat transthoracic echocardiography showed preserved left ventricular systolic function and no residual interatrial communication. Severe biatrial enlargement with spontaneous echo contrast persisted. The right ventricle remained dilated with reduced systolic function, with a tricuspid annular plane systolic excursion (TAPSE) of 1.3 cm. Tricuspid regurgitation was moderate to severe, and estimated systolic pulmonary artery pressure was approximately 38 mmHg (Figure 5).

**Figure 5:**
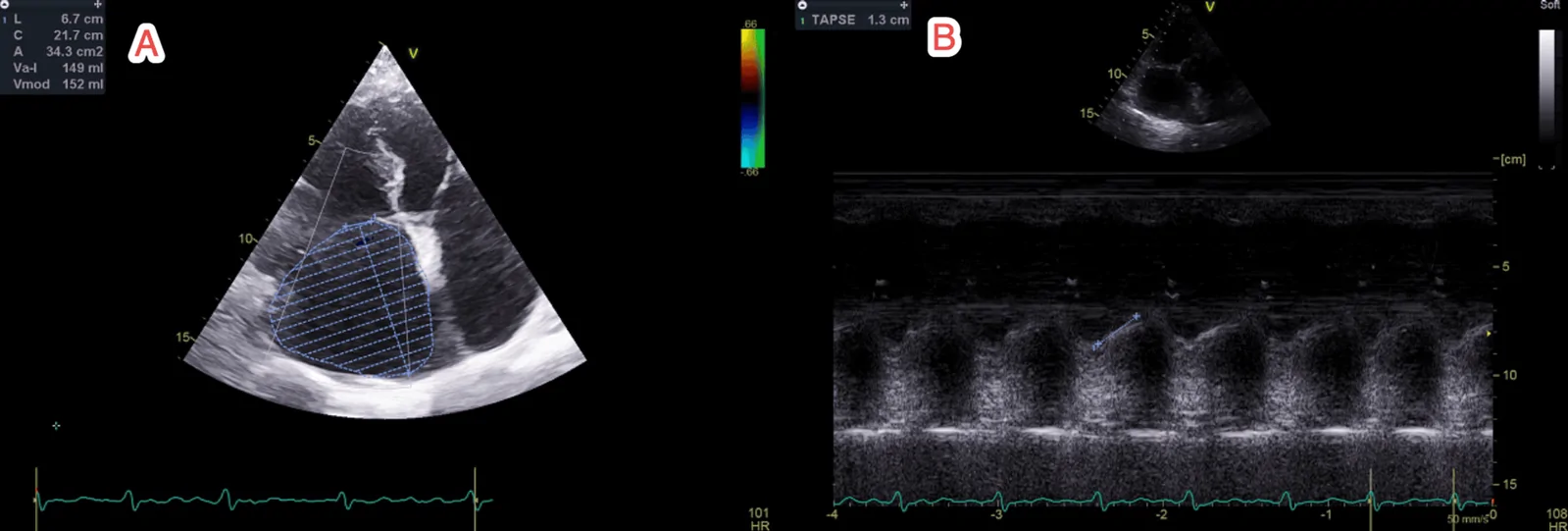
Postoperative transthoracic echocardiographic assessment of persistent right-sided remodeling and right ventricular systolic dysfunction. (A) Apical four-chamber view demonstrating marked right-sided chamber enlargement. The right atrial area measured 34.3 cm², with a calculated volume of approximately 149 mL and a modified volume measurement of approximately 152 mL. (B) M-mode assessment of tricuspid annular plane systolic excursion (TAPSE) showing a value of 1.3 cm (13 mm), consistent with reduced right ventricular longitudinal systolic function.

Agitated saline contrast testing on transthoracic echocardiography was negative, with no evidence of residual right-to-left interatrial shunting after surgical repair (Figure 6).

**Figure 6:**
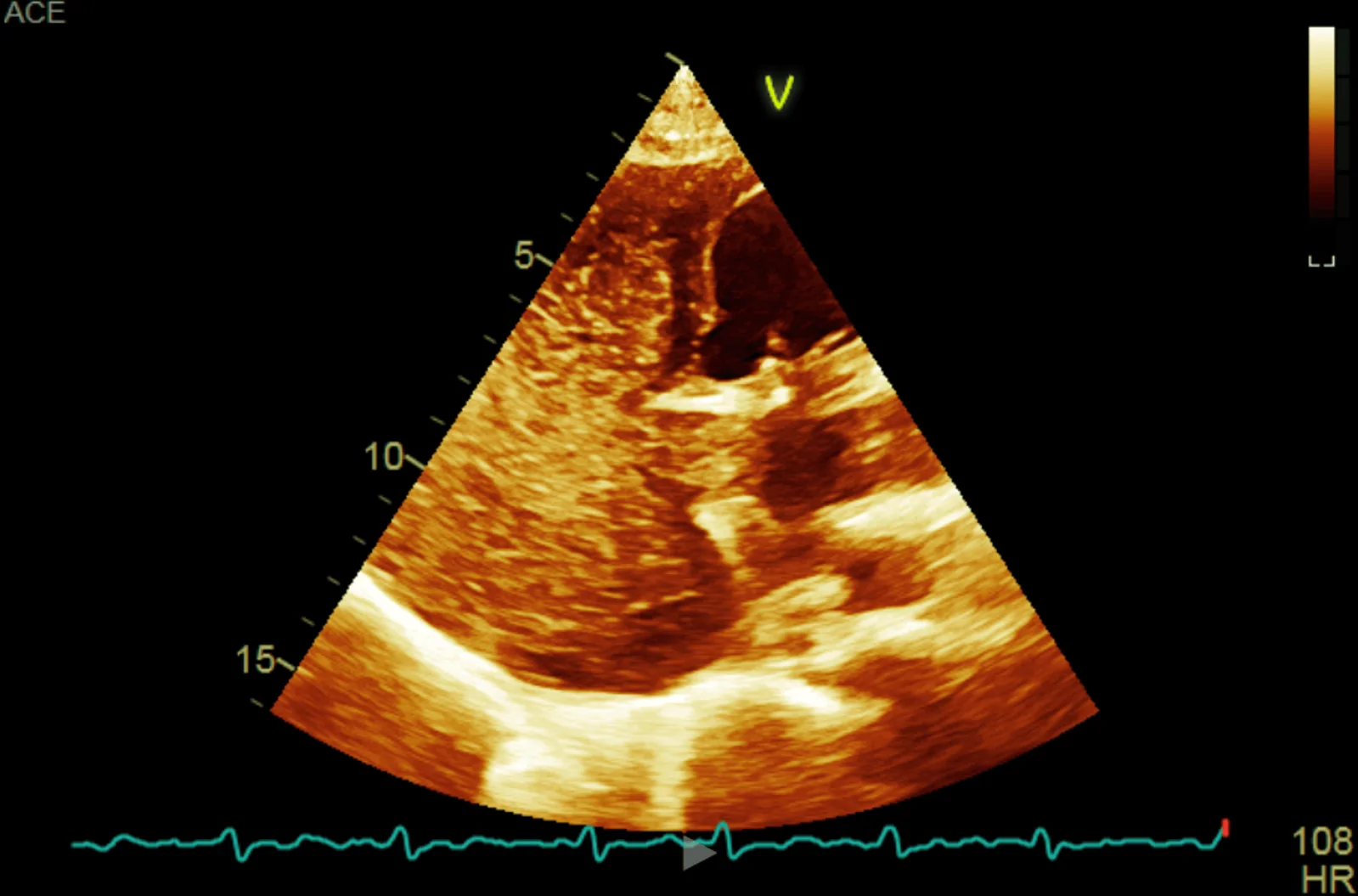
Negative transthoracic agitated saline contrast study after surgical repair.

Mitral regurgitation was mild to moderate. Tricuspid regurgitation had progressed to moderate to severe and remained functional in appearance. Mild aortic regurgitation was also present. The estimated systolic pulmonary artery pressure was approximately 38 mmHg.

Transesophageal echocardiography was then performed to further assess the repaired atrial septal region, evaluate the left atrial appendage, and search for an intracardiac thromboembolic source. The bicaval view showed an intact surgical patch at the repaired superior atrial septal region, with no evidence of patch dehiscence. The left atrial appendage was free of thrombus. Spontaneous echo contrast was again observed in both atria, consistent with atrial stasis, but no thrombus was identified in the left atrium, right atrium, or left atrial appendage (Figure 7).

**Figure 7:**
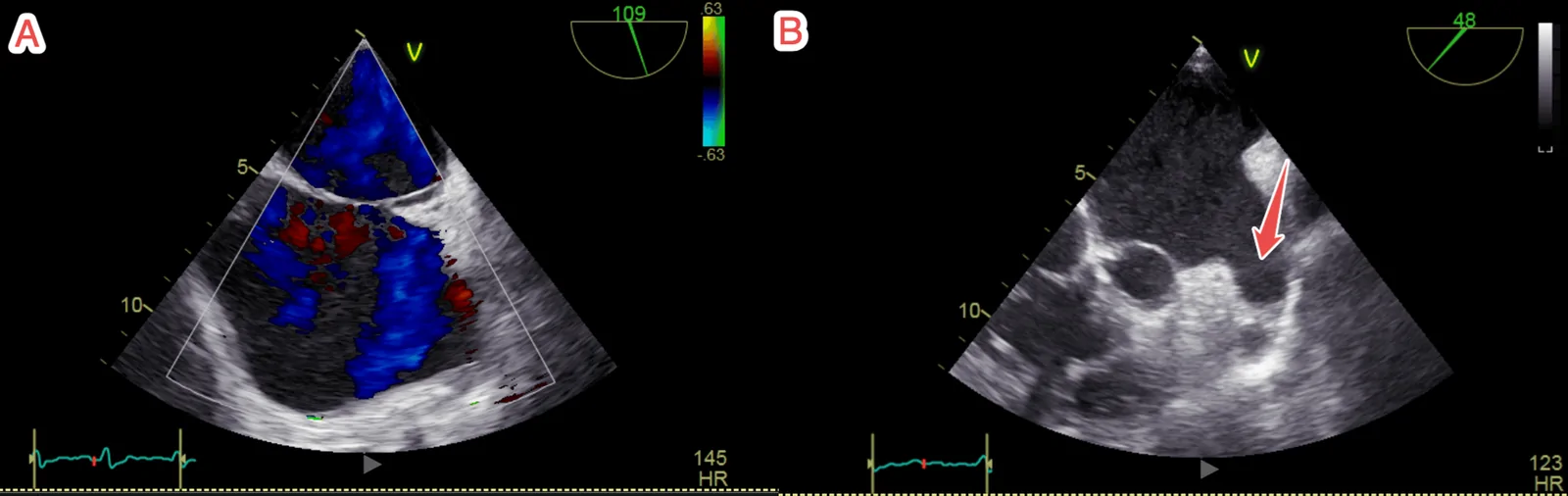
Postoperative transesophageal echocardiographic assessment of the repaired atrial septum and left atrial appendage. (A) Bicaval transesophageal echocardiographic view showing the repaired superior atrial septal region with the surgical patch intact and no evidence of dehiscence. (B) Transesophageal echocardiographic view showing an empty left atrial appendage with no thrombus.

Given the recent cardiac operation and the neurologic presentation, infective endocarditis was also considered as a potential source of systemic or cerebral embolization. Three sets of blood cultures were obtained and remained negative. Transesophageal echocardiography showed no valvular vegetation or other echocardiographic evidence of infective endocarditis.

Because the episode of loss of consciousness could not be fully explained by the cardiac findings, neurologic evaluation was pursued. Brain magnetic resonance imaging demonstrated diffuse abnormal signal involving the supratentorial cerebral cortex, predominantly within the right hemisphere. The frontal, parietal, and temporal cortices were affected. Abnormal signal also involved the caudate nuclei, predominantly on the right, and the right putamen, with less extensive involvement of the left medial parietal cortex.

The abnormalities were hyperintense on diffusion-weighted and fluid-attenuated inversion recovery sequences. An axial diffusion-weighted image clearly demonstrated abnormal signal involving the right putamen (Figure 8).

**Figure 8:**
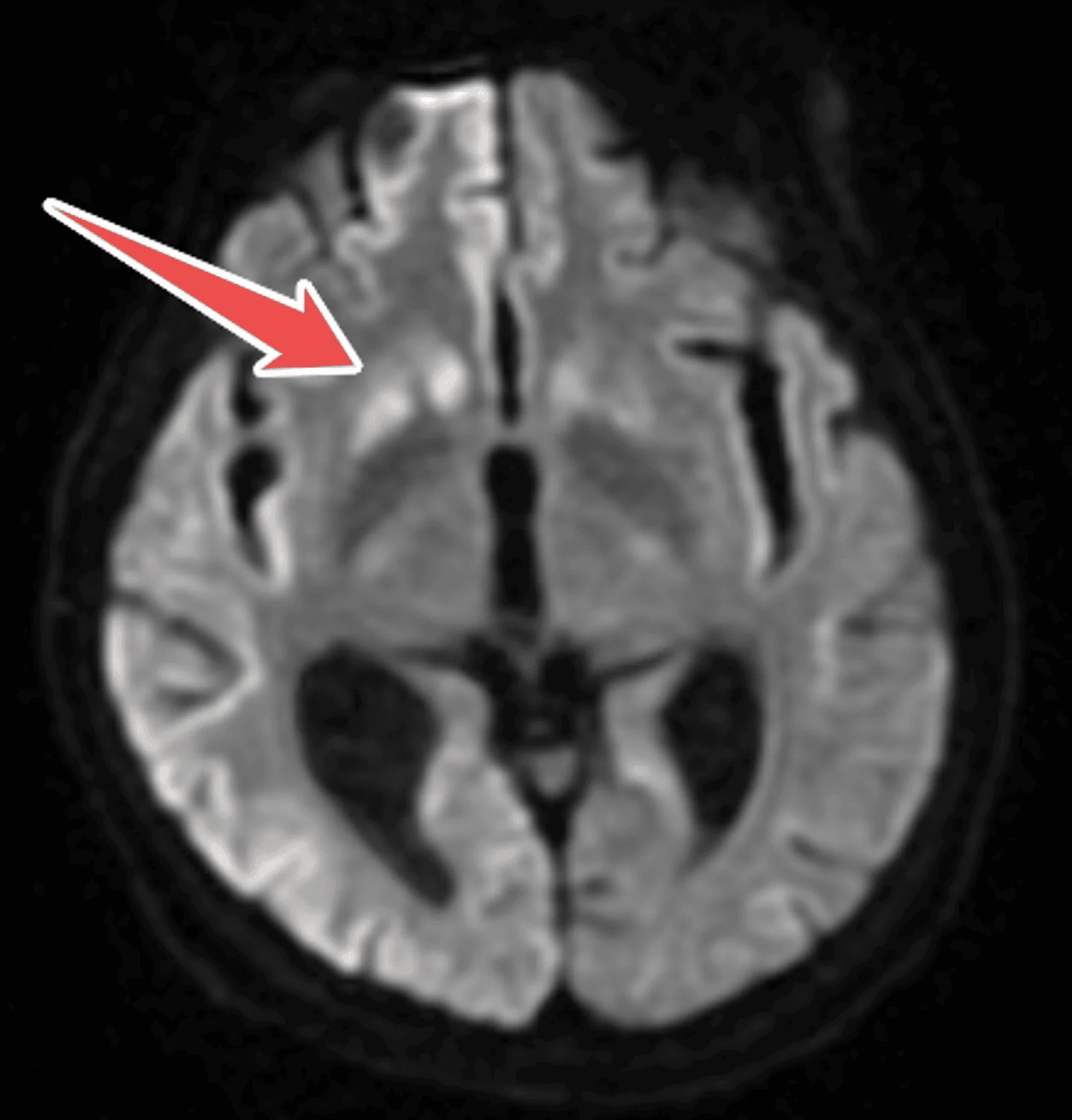
Axial diffusion-weighted brain MRI showing right putaminal hyperintensity, as highlighted by an arrow.

The pattern raised consideration of ischemic, inflammatory, infectious, metabolic, and seizure-related processes. There was no intracranial hemorrhage, hydrocephalus, or midline shift. Intracranial and extracranial magnetic resonance angiography showed no significant stenosis or abnormal vascular enhancement, although interpretation was partially limited by motion artifacts.

Electroencephalography was normal. Background activity was appropriately organized and reactive, with no focal slowing, epileptiform discharges, or electrographic seizure activity. A normal routine electroencephalogram did not support an ongoing epileptiform process at the time of recording, although it could not completely exclude a transient preceding seizure.

Because the brain magnetic resonance imaging remained abnormal despite the normal electroencephalogram, the evaluation was broadened toward infectious, inflammatory, autoimmune, and metabolic causes. The autoimmune and thromboinflammatory workup, including anti-double-stranded DNA antibodies, antiphospholipid antibodies, anticardiolipin antibodies, and anti-NMDA receptor antibodies, was negative.

Lumbar puncture was planned to investigate the neurologic abnormalities. Apixaban was temporarily interrupted to permit lumbar puncture, with periprocedural anticoagulation managed by the treating team. The cerebrospinal fluid was clear and colorless, with an opening pressure of 18 cm H₂O. The complete findings are presented in Table 2.

**Table 2:** Cerebrospinal fluid findings during the neurologic admission. CSF: cerebrospinal fluid; PCR: polymerase chain reaction; HSV: herpes simplex virus; VZV: varicella-zoster virus

Parameter	Result	Reference range
Appearance	Clear and colorless	Clear and colorless
Opening pressure	18 cm H₂O	6-25 cm H₂O
White blood cells	85 cells/mm³	0-5 cells/mm³
Differential count	90% lymphocytes, 10% neutrophils	No pleocytosis; predominantly lymphocytes/monocytes if present
Red blood cells	2 cells/mm³	0 cells/mm³ or absent
Protein	62 mg/dL, mildly elevated	15-45 mg/dL
Glucose	68 mg/dL	50-80 mg/dL or >50% of serum glucose
Simultaneous serum glucose	100 mg/dL	Paired serum value used to interpret CSF glucose
CSF/serum glucose ratio	0.68, preserved	Approximately ≥0.6
Gram stain	No organisms seen	No organisms seen
Bacterial culture	No growth	No growth
Fungal culture	No growth	No growth
Meningitis/encephalitis PCR panel	Enterovirus detected	Not detected
HSV-1/HSV-2 PCR	Not detected	Not detected
VZV PCR	Not detected	Not detected

The cerebrospinal fluid showed lymphocytic pleocytosis, mildly elevated protein, preserved glucose and CSF-to-serum glucose ratio, and negative Gram stain and bacterial and fungal cultures. Enterovirus was detected on the meningitis/encephalitis polymerase chain reaction panel. HSV-1, HSV-2, and varicella-zoster virus polymerase chain reaction testing were negative. The combined cerebrospinal fluid findings were compatible with enteroviral meningitis. The associated cortical and deep gray matter MRI abnormalities and transient alteration of consciousness raised concern for probable mild meningoencephalitic involvement despite the normal electroencephalogram. The patient received supportive treatment, including hydration according to clinical need, antiemetic therapy when required, and neurologic monitoring with serial assessment of consciousness and surveillance for seizures. No specific antiviral therapy was administered.

Abdominopelvic computed tomography showed mild periportal edema. Hepatitis testing was negative, and in the context of persistent right atrial enlargement, reduced right ventricular systolic function, moderate to severe tricuspid regurgitation, and an NT-proBNP level of 3,897 pg/mL, the finding was considered most consistent with hepatic venous congestion related to persistent right-sided hemodynamic stress.

The differential diagnosis for the transient loss of consciousness included arrhythmic and conduction disturbances, postoperative structural complications, thromboembolic cerebral ischemia, seizure, metabolic or autoimmune encephalopathy, and central nervous system infection. Cardiac monitoring and postoperative imaging did not identify a definitive cardiac cause. The positive enterovirus PCR, inflammatory cerebrospinal fluid profile, and abnormal brain MRI supported enteroviral meningitis with probable mild meningoencephalitic involvement as the most likely infectious diagnosis, although the mechanism of the loss of consciousness remained uncertain.

At one-month follow-up, the patient was clinically well. There was no recurrence of loss of consciousness, no new neurologic symptoms, and no deterioration in functional status.

## Discussion

This case illustrates two clinically relevant aspects of late-detected SVASD. First, superior SVASD with associated PAPVR may remain undetected on routine transthoracic echocardiography despite marked right-sided enlargement, making multimodality imaging important when an unexplained right-sided volume overload is present [1-5].

CMR and right heart catheterization confirmed a large left-to-right shunt, with Qp/Qs values of 3.7 and 3.2, respectively, and a low indexed pulmonary vascular resistance of 1.7 Wood units·m², supporting surgical repair [1,2,6]. The modest difference between the two Qp/Qs measurements was compatible with the different measurement techniques and did not alter the clinical interpretation [7].

Following repair, the patient had no residual interatrial shunt but showed persistent right ventricular dilatation and systolic dysfunction with progression of functional tricuspid regurgitation. Incomplete right ventricular reverse remodeling may occur after closure of longstanding atrial-level shunts, particularly in older patients [8-10].

When transient loss of consciousness occurred three and a half months later, postoperative cardiac causes remained important considerations, including atrial arrhythmias, conduction disturbances, residual shunting, and venous pathway complications [11]. However, inpatient monitoring showed no sustained ventricular arrhythmia, high-grade atrioventricular block, or significant pauses, and postoperative imaging showed no residual interatrial shunt. The absence of a definitive cardiac explanation prompted evaluation for alternative neurologic and infectious causes.

Given the patient's atrial fibrillation and marked biatrial enlargement with spontaneous echo contrast, thromboembolic cerebral ischemia was an important competing diagnosis. Diffusion-weighted hyperintensity can occur in acute ischemia, and the absence of a visible left atrial appendage thrombus on transesophageal echocardiography does not by itself exclude a preceding embolic event. However, the MRI abnormalities involved multiple cortical and deep gray matter regions rather than demonstrating a single straightforward vascular-territory lesion, and magnetic resonance angiography showed no significant stenosis or abnormal vascular enhancement. The imaging pattern therefore prompted consideration of ischemic, inflammatory, infectious, metabolic, and seizure-related causes.

Infective endocarditis was also considered because of the recent cardiac operation and the possibility of neurologic manifestations related to septic embolization. Three sets of blood cultures remained negative, and transesophageal echocardiography showed no valvular vegetation or other echocardiographic evidence of infective endocarditis. These findings made infective endocarditis less likely.

The cerebrospinal fluid findings subsequently provided evidence of a viral central nervous system infection. Viral meningitis typically produces clear cerebrospinal fluid, a normal or mildly elevated opening pressure, lymphocytic pleocytosis, preserved glucose, and normal or mildly elevated protein [12-14]. In this patient, the cerebrospinal fluid contained 85 white blood cells/mm³ with 90% lymphocytes, mildly elevated protein of 62 mg/dL, and a preserved CSF-to-serum glucose ratio of 0.68, while bacterial and fungal cultures were negative. Molecular testing of cerebrospinal fluid has an important role in identifying viral central nervous system infections [15]. Enterovirus was detected by polymerase chain reaction, and, together with the compatible inflammatory cerebrospinal fluid profile, this supported enteroviral central nervous system infection.

The normal electroencephalogram showed no focal slowing, epileptiform discharges, or electrographic seizures. Although a normal routine electroencephalogram cannot completely exclude a transient preceding seizure, it provided no evidence of an ongoing epileptiform process at the time of recording. The cortical and deep gray matter MRI abnormalities, together with the transient alteration of consciousness, raised the possibility of parenchymal involvement beyond an isolated meningeal syndrome. In the context of the positive enterovirus polymerase chain reaction (PCR) and inflammatory cerebrospinal fluid profile, enteroviral meningitis with probable mild meningoencephalitic involvement was considered the most likely infectious diagnosis. Nevertheless, the precise mechanism of the transient loss of consciousness remained uncertain, and a preceding seizure or ischemic component could not be excluded with certainty.

The autoimmune evaluation, including anti-NMDA receptor antibodies, anti-double-stranded DNA antibodies, antiphospholipid antibodies, and anticardiolipin antibodies, was reasonable before an infectious cause had been established. Autoimmune encephalitis can present with altered consciousness, seizures, cortical abnormalities, and focal or diffuse EEG changes. In this case, the positive enterovirus PCR and compatible cerebrospinal fluid (CSF) inflammatory profile offered a more direct explanation for the acute neurologic presentation.

Most uncomplicated enteroviral meningitis is treated supportively. Hydration, analgesic and antipyretic therapy, antiemetics when needed, and close neurologic observation for seizures or deterioration in consciousness are appropriate components of management [12-14]. The patient improved without specific antiviral therapy and remained clinically well at one-month follow-up.

The broader teaching point is the risk of diagnostic anchoring after cardiac surgery. This patient had several plausible cardiac explanations for transient loss of consciousness, including atrial fibrillation, postoperative conduction disease, right ventricular dysfunction, and evolving tricuspid regurgitation. A parallel neurologic evaluation identified enteroviral central nervous system infection, although the precise relationship between the infection and the transient loss-of-consciousness episode could not be established with certainty. New symptoms after congenital heart surgery should therefore be evaluated on their own merits rather than automatically attributed to the repaired cardiac lesion.

## Conclusions

This case highlights the diagnostic challenge of transient loss of consciousness occurring three and a half months after repair of a late-detected SVASD with partial anomalous pulmonary venous return. Cardiac, thromboembolic, infectious, and neurologic causes were investigated, and cerebrospinal fluid findings supported enteroviral central nervous system infection, although its precise relationship to the transient loss of consciousness remained uncertain. New neurologic symptoms after congenital cardiac surgery should therefore be evaluated broadly and should not automatically be attributed to the underlying cardiac condition or its surgical repair.
